# Temperature Regulation of Primary and Secondary Seed Dormancy in *Rosa canina* L.: Findings from Proteomic Analysis

**DOI:** 10.3390/ijms21197008

**Published:** 2020-09-23

**Authors:** Tomasz A. Pawłowski, Barbara Bujarska-Borkowska, Jan Suszka, Tadeusz Tylkowski, Paweł Chmielarz, Ewelina A. Klupczyńska, Aleksandra M. Staszak

**Affiliations:** Institute of Dendrology, Polish Academy of Sciences, Parkowa 5, 62-035 Kórnik, Poland; bbujarska@man.poznan.pl (B.B.-B.); jsuszka@man.poznan.pl (J.S.); ttylkows@man.poznan.pl (T.T.); pach@man.poznan.pl (P.C.); evelin@man.poznan.pl (E.A.K.); staszak.a@gmail.com (A.M.S.)

**Keywords:** dormancy, gene expression, germination, horticulture, proteins, reproduction, rose, seed

## Abstract

Temperature is a key environmental factor restricting seed germination. Rose (*Rosa canina* L.) seeds are characterized by physical/physiological dormancy, which is broken during warm, followed by cold stratification. Exposing pretreated seeds to 20 °C resulted in the induction of secondary dormancy. The aim of this study was to identify and functionally characterize the proteins associated with dormancy control of rose seeds. Proteins from primary dormant, after warm and cold stratification (nondormant), and secondary dormant seeds were analyzed using 2-D electrophoresis. Proteins that varied in abundance were identified by mass spectrometry. Results showed that cold stratifications affected the variability of the highest number of spots, and there were more common spots with secondary dormancy than with warm stratification. The increase of mitochondrial proteins and actin during dormancy breaking suggests changes in cell functioning and seed preparation to germination. Secondary dormant seeds were characterized by low levels of legumin, metabolic enzymes, and actin, suggesting the consumption of storage materials, a decrease in metabolic activity, and cell elongation. Breaking the dormancy of rose seeds increased the abundance of cellular and metabolic proteins that promote germination. Induction of secondary dormancy caused a decrease in these proteins and germination arrest.

## 1. Introduction

Temperature has a major influence on seed dormancy and germination, as it is one factor coordinating plant development with climate variability [1]. Temperature signaling is transduced to plant physiology and genetics in a multidimensional manner [1]. Seed dormancy is an evolutionary, environmentally imprinted adaptive trait that prevents germination under unfavorable temperature conditions. If seeds able to germinate lose this ability due to stressful environmental conditions (e.g., too high temperatures), secondary dormancy is established [2]. Such seeds revert to dormancy and overlap until the following spring or longer, which is a beneficial process from a biological standpoint (seed banks) but undesirable from an economic one, e.g., in a nursery. For their germination, repeated stratification is needed, but this does not guarantee eventual success. Finch–Savage and Leubner–Metzger [3] suggested that primary dormancy correlates to slow seasonal change (temporal sensing), associated with cycling from deep to shallow dormancy to select the climate space for emergence and time of year. Seasonal temperature patterns regulate the cycle. Secondary dormancy correlates with a rapid response to the suitability of local conditions for germination and plant establishment (spatial sensing). Under natural conditions, such environment-controlled dormancy is manifested as dormancy cycling between seasons and years [4].

Germination cueing can be a specified form of phenological cueing, since some environmental conditions must appear to break dormancy, and additional environmental conditions must occur to enable germination after dormancy is broken [5]. When seeds lose dormancy, the range of environmental conditions in which they can germinate broadens, and as secondary dormancy appears, that range narrows again [5]. Thus, the pace of primary dormancy loss, secondary dormancy introduction, and the attributes of temperature-dependent germination regulate not only the season but the long-term life history that is expressed.

For seeds of many plant species, the breaking of dormancy is not a quick change between dormant and nondormant states, but a series of continuous changes in the entire seed to the molecular level, from complete dormancy to complete nondormancy [6]. How this complicated system is used by the seed to adjust dormancy cycling in fluctuating environments is still unknown [7]. Information regarding seed dormancy and annual seasonal changes in the germination capacity of seeds may be advantageous to researchers in determining the best study pathways.

Physiological, molecular, and genetic analyses have provided insights into the mechanisms of seed dormancy and germination [8,9]. Plant hormones, including ABA, GA, auxin, or ethylene, are implicated in the regulation of dormancy status, including secondary dormancy [10,11,12,13,14,15,16]. The results of Footitt et al. [4] indicated that soil temperatures trigger seed-specific temporal sensing via the accumulation of DOG1 protein to drive changes in germination potential. Dormancy cycling is regulated by clock genes and the dormancy-related genes *DOG1*, mother of flowering time (*MFT)*, CBL-interacting protein kinase 23 (*CIPK23*), and phytochrome A (*PHYA*) [17]. *DOG1* participates in the induction of primary dormancy in response to cold maturation temperature acting on maternal plants and also participates in secondary dormancy in response to warm and cold stratification [5,18]. As *DOG1*-imposed dormancy alters responses to germination temperatures, *DOG1* strongly influences the environmental responsiveness of germination. Chiang et al. [19] considered the timing of germination, and particularly *DOG1*-controlled dormancy, to be associated with life-history alteration.

Wild rose seeds are characterized by the occurrence of combinational dormancy, which consists of physiological dormancy of the embryo and physical dormancy associated with seed coat properties [20]. This type of physical/physiological dormancy is broken during warm, followed by cold stratification (25 °C/3 °C) lasting several months [21]. A temperature of 20 °C used on pretreated seeds caused the induction of secondary dormancy. Hilhorst [22] characterized secondary dormancy as a phenomenon that occurs after seed dispersal and the loss of primary dormancy. Edwards et al. [23] suggested that seeds must have some degree of primary dormancy to be capable of entering secondary dormancy. 

In this study, we investigate how temperature affects proteome changes in *Rosa canina* seeds during primary dormancy release by warm and cold stratification and secondary dormancy induction by warm treatment. We hypothesized that there are certain similarities between the regulation of primary and secondary dormancy of seeds. One goal of the present study is to reveal differentially abundant proteins to identify those putatively responsible for the breaking of primary dormancy and induction of secondary dormancy of rose seeds.

## 2. Results and Discussion

**Figure 1:**
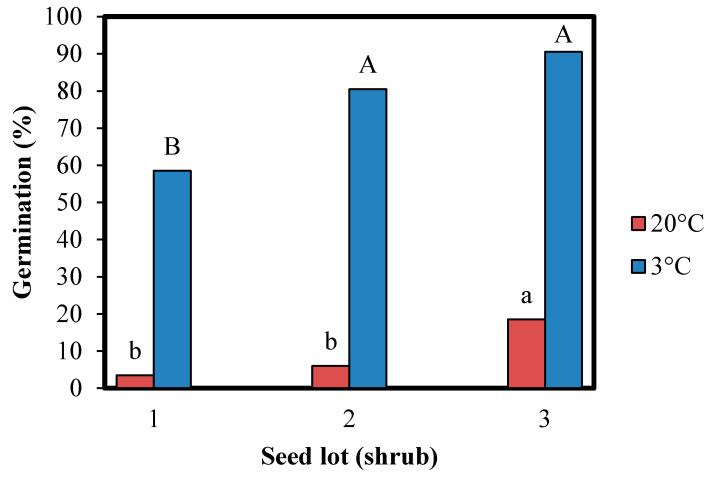
Effect of temperature on *Rosa canina* L. seed germination after dormancy breaking by warm/cold stratification (16 weeks at 25 °C followed by 22 weeks at 3 °C). Germination tests were performed for 8 weeks at 3 and 20 °C. Seeds were collected from three different shrubs (1–3). Data with different letters (lowercase for 20 °C and capital for 3 °C) are significantly different, *p* < 0.05 (ANOVA and a Tukey–Kramer HSD).

The dormancy cycling phenomenon has been widely studied, but the molecular mechanism responsible remains largely unknown. Recent transcriptomic studies indicate that seeds vary and remain active at a molecular level in both primary and secondary dormancy [13,16,24,25,26]. Despite the many studies of primary dormancy proteomics, not one has considered secondary dormancy. In the present research, the main focus was on secondary dormancy to reveal proteins responsible for its control. We hypothesized, however, that there are certain similarities between the regulation of primary and secondary dormancy of seeds. Wild rose seeds were chosen as an object of study because of the practical difficulty in seed germination and emergence in field conditions corresponding to the undesirable trait of warm temperature-induced secondary dormancy. We observed that rose seed combinational dormancy was broken during warm (25 °C, 16 weeks) followed by cold stratification (3 °C, 22 weeks). Pretreated seeds were exposed to a temperature of 20 °C to induce secondary dormancy, in contrast to 3 °C, which promoted germination (Figure 1). At 20 °C, seed germination for lot No. 1 reached only 3.5%, lot No. 2, 6.0%, and lot No. 3, 18.5%. Germination ability at 3 °C reached 58.5%, 80.5%, and 92, 0%, respectively. Seed germination was dependent on individual variability (seed lot origin from a different shrub). 

**Figure 2:**
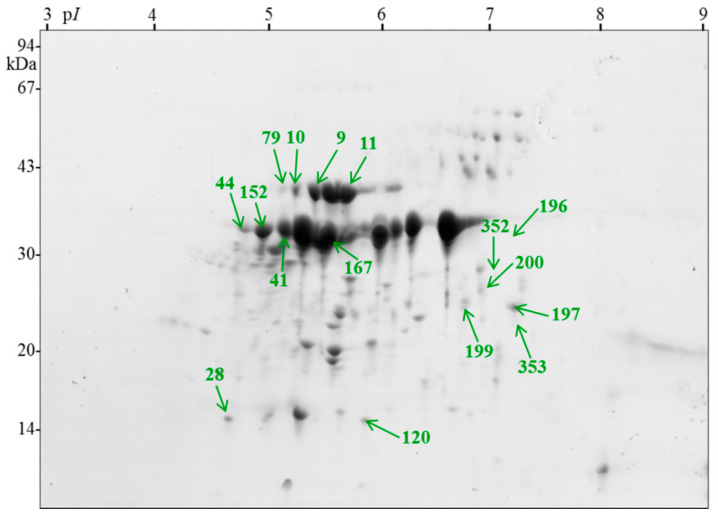
Reference gel demonstrating positions of statistically significantly variable spots from *Rosa canina* L. between dry seeds (primary dormant), seeds after warming, seeds after the cold phase of stratification (nondormant), and seeds after germination test at 20 °C (secondary dormant). The numbers of identified proteins correspond to those listed in Table 1.

For further proteomic investigations, seeds were taken from three seed lots from four time points: dry seeds (primary dormant), seeds after the warm phase of stratification, seeds after the cold phase of stratification (nondormant seeds), and seeds after germination testing at 20 °C with induced secondary dormancy (Figure S1). An average of 543 Coomassie blue-stained spots were detected on each two-dimensional gel representing each sample using the Image Master 7 Platinum program. A total of 16 spots exhibiting significant changes in abundance for temperature and origin, representing approximately 3% of the total number of spots on a reference gel (Figure 2) were identified by MS (Table 1). 

As listed in Table 1, all 16 spots represented 14 nonredundant proteins. The various spots identified as the same protein (spots 41 and 167 as well as 199 and 200) could correspond either to post-translational modification (PTM) of the same protein or to various isoforms. The percentage of sequence coverage ranged from 5 to 46%, and the number of identified peptides varied from 1 to 40. Among the 16 spots, four corresponded to the *Rosa* genus, three to *Fragaria vesca* (Rosaceae family), and nine to other plants. Homologous proteins were found for all of the spots. 

Among the 16 identified proteins, 12 showed variability in abundance for temperature (Table 2), six for origin, and two for both (ANOVA, the Tukey–Kramer HSD test, *p* < 0.05). The temperature-induced secondary dormancy of rose seeds had an impact on the variability of six proteins compared to control, two proteins compared to warm stratification, and one protein compared to cold stratification (Table 2). Eight proteins were variable for cold stratification compared to dry seeds, and two proteins compared to warm stratification. Five proteins were variable for warm stratification compared to dry seeds (Table 2). 

Here, we discuss the role of the proteins (due to the function and their related metabolic pathways) that can contribute to the dormancy status of the seeds in relation to system biology approaches [28].

Seed dormancy breaking and germination is a multifaceted process, associated with changes in the gene expression, protein synthesis, and physiology, but also with organelle functioning [29,30,31]. Numerous mitochondrial proteins have been identified as being involved in respiration, tricarboxylic acid (TCA) cycle, metabolism, import, and stress response as potentially important for seed germination [32,33]. Eight rose seed proteins identified in the present study (they are involved in glycolysis, TCA cycle, ATP synthesis, translation, stress response, cell activity, and transport) were predicted to be localized in mitochondria. This data confirms the link between mitochondrial functioning and the regulation of seed germination. 

Legumin B-like protein (spots 167 and 41, decreased during secondary dormancy in comparison to dry seeds and cold stratification, respectively) is a plant seed storage protein, whose dominant function appears to be as a major nitrogen source for the developing plant [34]. Legumins accumulate gradually throughout maturation concomitantly with ABA [35]. They are present in dry seeds, essentially in cotyledons and hypocotyls, but disappear during their germination [36,37]. The decrease of this protein observed in the present study indicates the consumption of storage materials necessary for seed dormancy breaking and germination and also for the initiation of secondary dormancy. Consumption of storage materials can cause difficulty in the breaking of rose seed secondary dormancy. 

Actin-7 (spot 79, increased during primary dormancy breaking in comparison to dry seeds) is associated with the regulation of hormone-induced plant cell proliferation and callus formation [38]. Accumulation of actin-7, as well as legumin, corresponded with the reserve deposition phase in *Medicago truncatula* seeds [39]. Actins, including actin-7, play an essential role in germination and root growth [40,41,42,43,44]. Actin-depolymerizing factor 2 (ADF2) proteins involved in a dynamic change in the cytoskeleton necessary for embryo cell elongation increased in abundance in germinated seeds but not in ungerminated thermoinhibited seeds [45]. The increase in abundance of actin-7 was observed in rose seeds after warm and cold stratification, proving its role in germination. Entrance into secondary dormancy decreased actin-7 abundance, likely suggesting its depolymerization and inhibition of cell elongation. 

Mitochondrial ADP/ATP translocator (ADP/ATP carrier 3, AAC3 *Arabidopsis* homolog, spot 152, increased during primary dormancy breaking in comparison to dry seeds) catalyzes the exchange of cytosolic ADP with matrix ATP across the mitochondrial membrane and, thus, enables the mitochondria to supply energy to the cytosol, and subsequently to other organelles [46]. Adenine nucleotide transporters were indicated to be crucial for growth, as well as for photorespiratory metabolism, and accumulation of proteins and storage lipids [47,48,49]. The in silico analysis of the expression of adenine nucleotide carriers showed that they are variable under various stress conditions [47]. The *AAC3* expression under stress conditions corresponds to genes associated with processes that rely on ATP-dependent enzymes of protein degradation pathways, such as ubiquitin-associated proteins [47]. A decrease in *AAC3* expression was indicated after seed imbibition; however, it reached a higher level in germinated seeds and seedlings [47,50,51]. The role of ADP/ATP carriers in seed germination can be associated with the change of quiescent mitochondria into active forms by providing ATP for actin activity [52]. 

The ATPase alpha subunit (ATPA, spot 197) forms a catalytic core of ATP synthase, which synthesizes ATP from ADP [53]. Energy in the form of ATP is needed for seed germination because germinating seeds lack both mineral uptake and photosynthetic systems [54]. The expression trend of the ATP synthase beta subunit showed an up-regulated pattern and demonstrated that energy metabolism continuously bolstered the process of germination [55]. The increase in activity of ATPases was observed during dormancy breaking of several tree seeds caused by cold stratification [40,56,57,58]. The present study demonstrated that the ATPase alpha subunit was up-accumulated during dormancy release of rose seeds, but this was observed only during warm stratification. Similarly, accumulation of triosephosphate isomerase (spots 199 and 200), a key enzyme in glycolysis, was shown to increase only during the warm phase of primary dormancy breaking. It seems that these enzymes are crucial for the warm phase of dormancy breaking because they provide adequate cellular ATP and carbohydrate metabolism levels. 

Elongation factor E1 (EF E1, spot 11) participates in the process of mitochondrial biogenesis and has been stress-induced by salinity in lupine embryos [59]. Alterations in the accumulation level of mitochondrial elongation factor Tu (homolog of EF E1) were observed in *Glycine max* seeds during imbibition [60]. The changes in the abundance of elongation factors were indicated prior to seed germination and were associated with the beginning of mitotic activity [61]. High expression of EF is necessary for the preservation of rapid protein synthesis and cell division in meristematic tissues, which is fundamental for seed dormancy breaking and germination [61]. EF E1 accumulated in rose seeds during primary dormancy breaking and induction of secondary dormancy on a similar level, suggesting its general engagement in protein synthesis.

## 3. Materials and Methods 

### 3.1. Plant Material 

Fully maturated seeds in nuts of the wild rose (*Rosa canina* L.) were collected in November 2015 from two proveniences: in Kobylepole near Poznań (lots no. 1 and 2 from two different shrubs, Poland, 52°23′ N and 17°01′ E) and in Pokrzywno (lot no. 3 from one shrub, Poland, 52°21′ N and 16°58′ E). The seeds were separated from the fruit and dried for 10 days at room temperature to 9% moisture content. For further experiments, intact seeds were collected.

### 3.2. Seed Germination

Seed stratification was started in November 2015 (Figure S1). The stratification substrate was composed of quartz sand and peat (pH 5.5–6.5). During stratification, the water content of seeds and substrate was controlled every week to aerate the seeds and replenish water losses. After 16 weeks of the warm phase (25 °C), seeds were transferred to the cold phase (3 °C) for 22 weeks. Those treatments break dormancy and promote seed germination. Subsequently, one portion of stratified seeds was subjected to the germination test at 3 °C temperature, which promotes seed germination. For the second portion the germination test was performed at 20 °C, a temperature which induces secondary dormancy. Germination tests were performed in 4 replicates of 50 seeds each for 8 weeks. Analysis of variance (ANOVA) and a Tukey–Kramer HSD were used to assess the influence of temperature on the level of seed germination, at *p* < 0.05 (JMP software, SAS Institute, Cary, NC, USA).

For further proteomic investigations, seeds were collected from three seed lots from four time points: dry seeds (primary dormant), seeds after the warm phase of stratification, seeds after the cold phase of stratification (nondormant seeds), and seeds after germination testing at 20 °C with induced secondary dormancy.

### 3.3. Proteome Analysis

The seeds were mechanically crushed and ground into powder in a mortar cooled with liquid nitrogen. Proteins of powdered seeds were precipitated for 1 hour at −20 °C in a 10% (*w/v*) solution of trichloroacetic acid (TCA) in acetone containing 20 mM dithiothreitol (DTT) [57]. After centrifugation and vacuum drying, the resulting pellets were resuspended in lysis buffer (7 M urea, 2 M thiourea, 2% (*w/v*) 3-([3-cholamidopropyl] dimethylammonio)-1-propanesulfonate (CHAPS), 1.5% (*w/v*) DTT, 0.5% (*v/v*) immobiline polyacrylamide gel (IPG) buffer pH 3–10), supplemented with a protease inhibitor cocktail (Roche, Basel, Switzerland). Protein concentrations were determined using the Bradford assay [62]. Three replicates of 60 seeds were analyzed for each time point and seed lot.

Proteins were first separated electrophoretically on immobiline dry strips (24 cm, pH 3–10) using an Ettan IPGphor 3 IEF System (GE Healthcare, Little Chalfont, UK) according to the manufacturer’s instructions. The strips were then equilibrated with solution I (6 M urea, 1.5 M Tris–HCl, pH 8.8, 30% (*v/v*) glycerol, 2% (*w/v*) SDS, 1% (*w/v*) DTT) and solution II (solution I without DTT, supplemented with 2.5% (*w/v*) iodoacetamide). EttanDALT12.5% (*w/v*) polyacrylamide precast gels and Ettan DALT Six electrophoretic chamber (GE Healthcare, Little Chalfont, UK) were used for second-dimension electrophoresis (SDS–PAGE). Triplicate gels were run for every sample (biological replicates). After electrophoresis, the gels were stained with colloidal Coomassie blue [63], scanned, and analyzed using 2D Image Master 7 Platinum software (GE Healthcare, Little Chalfont, UK). After spot detection, 2D gels were aligned and matched, and normalized spot volumes were determined quantitatively. For each matched spot, the percent volume (abundance) was calculated as the volume divided by the total volume of matched spots. The spots showing variations in abundance were subjected to ANOVA and a Tukey–Kramer HSD test (JMP software, SAS Institute, Cary, NC, USA) to select spots significantly variable in abundance during maturation (*p* < 0.05). These proteins were identified by mass spectrometry (MS).

Proteins were subjected to a standard “in-gel digestion” procedure [64]. Peptide mixtures were separated by liquid chromatography (LC) before molecular mass measurements (LC coupled to an LTQ-FTICR mass spectrometer) on an Orbitrap Velos mass spectrometer (Thermo Electron Corp., San Jose, CA, USA) at the Mass Spectrometry Laboratory (Institute of Biochemistry and Biophysics, Polish Academy of Sciences, Warsaw, Poland). A peptide mixture was applied to an RP-18 pre-column, then transferred to a nano-HPLC RP-18 column (Waters, Milford, MA, USA). The column outlet was directly coupled to the electrospray ionization (ESI) ion source of an Orbitrap Velos mass spectrometer (Thermo Electron Corp., San Jose, CA, USA), working in the regime of the data-dependent MS to MS/MS switch. An electrospray voltage of 1.5 kV was used. 

Acquired data were pre-processed with Mascot Distiller software (ver. 2.3.2.0, Matrix Science, London, UK), followed by a database search using the Mascot Search engine (Matrix Science, London, UK) against the NCBInr (National Centre for Biotechnology Information, Bethesda, MD, USA) database (ver. 20120224) with a Viridiplantae filter. The search parameters for precursor and product ion mass tolerance were 40 ppm and 0.6 Da, respectively. Protein identification was performed using the Mascot search probability-based molecular weight search (MOWSE) score. The ion score was -10 x log(P), in which P was the probability that the observed match was a random event. Peptides with a Mascot Score exceeding the threshold value corresponding to a < 5% false-positive rate were considered to be positively identified.

## 4. Conclusions

Proteomic analysis showed that the cold temperature-induced dormancy breaking of rose seeds had an impact on the variability of the highest number of spots, and had more common spots with secondary dormancy then with the warm stratification. We found that the proteins generally were up-accumulated during dormancy breaking, but they were down-regulated during secondary dormancy induction. Functional analysis of identified proteins showed that induction of secondary dormancy caused storage protein consumption and a decrease in abundance of actin and metabolism enzymes. This can cause difficulty in the breaking of rose seed secondary dormancy. Results of the present study provide valuable information, revealing the general regulation of significant proteins following the varying temperatures of seed primary dormancy breaking and secondary dormancy induction. Overall, this data should enhance understanding of the processes associated with seed dormancy. The highlighting of potentially important proteins by proteomics provides researchers with starting points for further studies where the next step will be to examine the expression and regulation of the gene encoding the protein of interest, to incorporate it into the seed dormancy level testing.

## Figures and Tables

**Table 1:** Identification of differentially abundant proteins of *Rosa canina* L. seeds during primary dormancy release by warm (25 °C) and cold (3 °C) stratification and secondary dormancy induction by warm treatment (20 °C).

Spot ^a^	Protein ^b^ [Species]	Accession ^c^	Theoretical		Experimental		Score	SC ^d^	All/No Repeat ^e^	Unic/No Repeat ^f^	emPAI ^g^
			**MW**	**p*I***	**MW**	**p*I***					
9	succinyl-CoA ligase beta subunit [*Arabidopsis thaliana*]	AAM65138.1	46	6.1	40	5.3	166	5	3/3	3/3	0.2
10	actin [*Lycoris longituba*]	AFP44112.1	42	5.3	41	5.3	751	29	11/9	0/0	1.72
11	elongation factor E1 [*Brassica oleracea var. capitata*]	AFL69959.1	49	6.1	40	5.3	725	21	10/9	9/8	0.98
28	temperature-induced lipocalin [*Solanum tuberosum*]	ABB02386.1	21	6.0	16	4.9	118	10	2/2	2/2	0.48
41	legumin B-like [*Fragaria vesca* subsp*. vesca*]	XP_004294115.1	57	6.8	36	5.2	483	16	31/6	16/3	0.56
44	adenosine kinase 2 [*Glycine soja*]	KHN02332.1	38	5.5	37	5	99	3	1/1	1/1	0.12
79	actin-7 [*Musa acuminata* subsp. *malaccensis*]	XP_009383456.1	42	5.3	41	5.2	350	16	5/5	0/0	0.65
120	cytosolic class I small heat-shock protein HSP17.5 [*Rosa* hybrid cultivar]	ABO84841.1	17	6.0	15	5.8	562	46	16/8	2/2	5.64
152	mitochondrial ADP/ATP translocator [*Chlamydomonas incerta*]	ABA01103.1	34	9.7	37	5.0	357	15	5/5	1/1	0.86
167	legumin B-like [*F. vesca* subsp*. vesca*]	XP_004294115.1	57	6.3	36	5.5	493	16	40/6	13/3	0.56
196	2-dehydro-3-deoxyphosphooctonate aldolase 1 [*F. vesca* subsp*. vesca*]	XP_004306551.1	32	6.6	32	6.1	590	33	9/8	9/9	2.24
197	ATPase alpha subunit, partial (mitochondrion) [*Chlorokybus atmophyticus*]	ABI54626.1	38	9.3	24	7	131	5	2/2	2/2	0.12
199	triosephosphate isomerase, cytosolic [*Zea mays*]	ACG24648.1	27	5.5	23	6.6	283	14	3/3	1/1	0.58
200	triosephosphate isomerase, cytosolic [*Z. mays*]	ACG24648.1	27	5.5	25	6.8	374	19	4/4	2/2	0.85
352	glyceraldehyde 3-phosphate dehydrogenase [*R. multiflora*]	AEQ75490.1	37	7.7	27	6.9	562	33	11/10	1/1	1.51
353	oil body-associated protein 1A-like [*R. chinensis*]	XP_024167493.1	27	5.9	26	7.1	408	19	6/5	5/4	1.19

^a^ The spot number is as indicated on the 2-D gels (Figure 2). ^b^ The proteins identified in the present study. Protein identification was based on the best hit in a MASCOT search against NCBI databases. ^c^ NCBI accession numbers. ^d^ Percentage of sequence coverage. ^e^ The number of all nonredundant peptides for each protein spot. ^f^ The number of unique to nonredundant sequences within a unique peptide number. ^g^ exponentially modified protein abundance index (emPAI) estimate the absolute protein amount in proteomics by the number of sequenced peptides per protein [27].

**Table 2:** The abundance of identified proteins of *Rosa canina* L. seed that significantly changed during primary dormancy release by warm (25 °C) and cold (3 °C) stratification and secondary dormancy induction by warm treatment (20 °C).

Spot ^a^	Protein ^b^	Mean % Volume (± s.d.) ^c^			
		Dry	Warm	Cold	Secondary
28	temperature-induced lipocalin	0.09 ± 0.07 c	0.22 ± 0.08 bc	0.39 ± 0.21 ab	0.46 ± 0.11 a
41	legumin B-like	1.16 ± 0.21 b	1.53 ± 0.30 b	2.25 ± 0.49 a	1.49 ± 0.38 b
79	actin-7	0.06 ± 0.01 b	0.12 ± 0.02 ab	0.20 ± 0.11 a	0.15 ± 0.05 ab
120	cytosolic class I small heat-shock protein HSP17.5	0.09 ± 0.01 b	0.08 ± 0.01 b	0.12 ± 0.06 ab	0.16 ± 0.03 a
152	mitochondrial ADP/ATP translocator	0.63 ± 0.16 b	0.85 ± 0.21 ab	1.15 ± 0.24 a	0.91 ± 0.20 ab
167	legumin B-like	4.96 ± 0.60 a	2.84 ± 0.82 ab	2.38 ± 1.11 b	2.23 ± 1.43 b
196	2-dehydro-3-deoxyphosphooctonate aldolase 1	0.08 ± 0.04 a	0.03 ± 0.04 ab	0 b	0 B
197	ATPase alpha subunit	0 b	0.11 ± 0.10 a	0.07 ± 0.10 ab	0.04 ± 0.06 ab
199	triosephosphate isomerase	0 b	0.05 ± 0.03 a	0.001 ± 0.001 b	0.02 ± 0.02 ab
200	triosephosphate isomerase	0 b	0.07 ± 0.02 a	0.05 ± 0.07 ab	0.06 ± 0.08 ab
352	glyceraldehyde 3-phosphate dehydrogenase	0.05 ± 0.04 a	0 b	0 b	0 B
353	oil body-associated protein 1A-like	0.13 ± 0.21 a	0 b	0 b	0 B

^a^ Spot number, as indicated on the reference gel (Figure 2). ^b^ The proteins identified in the present study. ^c^ The mean value with the standard deviation of six spot volumes at each analyzed stage: dry, stratified seeds at 25 (warm), and 3 °C (cold) and seeds being under secondary dormancy after germination test at 20 °C. Spots were subjected to ANOVA and Tukey–Kramer HSD test to select spots that significantly varied (*p* < 0.05) in abundance. Levels not represented by the same letter are significantly different.

## Supplementary Materials

Supplementary materials can be found at https://www.mdpi.com/1422-0067/21/19/7008/s1. Figure S1. Experimental design of *Rosa canina* L. seed dormancy breaking by warm/cold stratification (16 weeks at 25 °C followed by 22 weeks at 3 °C) and germination tests (8 weeks at 3 °C and 20 °C). P1-4 terms of sample collections.
