# Supplementary material for: Temperature Regulation of Primary and Secondary Seed Dormancy in Rosa canina L.: Findings from Proteomic Analysis
Source: Int J Mol Sci. 2020 Sep 23;21(19):7008. doi: 10.3390/ijms21197008 (PMC7582745; doi:10.3390/ijms21197008)
Supplement: Supplementary file 1 [file ijms-21-07008-s001.pptx]

## Slide 1
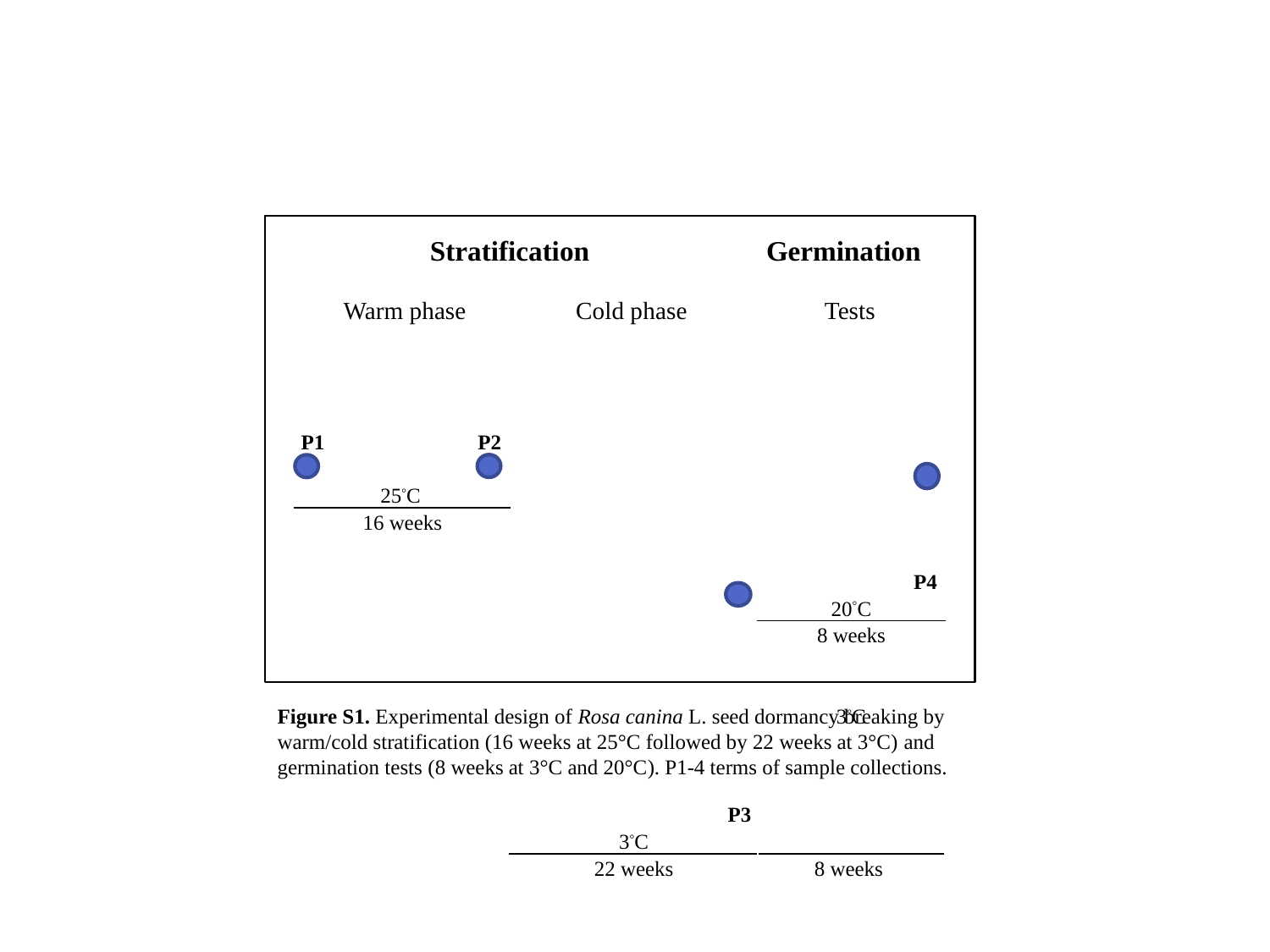

Stratification
Germination
| Warm phase | Cold phase | Tests |
| --- | --- | --- |
| P1 P2 25°C | P3 3°C | P4 20°C |
| --- | --- | --- |
| 16 weeks | | |
| | | 8 weeks 3°C |
| | 22 weeks | 8 weeks |
Figure S1. Experimental design of Rosa canina L. seed dormancy breaking by warm/cold stratification (16 weeks at 25°C followed by 22 weeks at 3°C) and germination tests (8 weeks at 3°C and 20°C). P1-4 terms of sample collections.
